# The Trans Golgi Region is a Labile Intracellular Ca^2+^ Store Sensitive to Emetine

**DOI:** 10.1038/s41598-018-35280-z

**Published:** 2018-11-21

**Authors:** Martín-Leonardo Gallegos-Gómez, Elisa Greotti, María-Cristina López-Méndez, Víctor-Hugo Sánchez-Vázquez, Juan-Manuel Arias, Agustín Guerrero-Hernández

**Affiliations:** 1 Department of Biochemistry, Cinvestav, Mexico City, 07000 Mexico; 2 Department of Biomedical Sciences, University of Padua, Padua, 35121 Italy; 3 Neuroscience Institute, Padova Section, National Research Council, Padua, 35121 Italy; 4 Programa de Neurociencias-UIICSE, Facultad de Estudios Superiores Iztacala, UNAM, Av. de los Barrios 1, Los Reyes Iztacala, 54090 Estado de México Mexico

## Abstract

The Golgi apparatus (GA) is a *bona fide* Ca^2+^ store; however, there is a lack of GA-specific Ca^2+^ mobilizing agents. Here, we report that emetine specifically releases Ca^2+^ from GA in HeLa and HL-1 atrial myocytes. Additionally, it has become evident that the trans-Golgi is a labile Ca^2+^ store that requires a continuous source of Ca^2+^ from either the external milieu or from the ER, to enable it to produce a detectable transient increase in cytosolic Ca^2+^. Our data indicates that the emetine-sensitive Ca^2+^ mobilizing mechanism is different from the two classical Ca^2+^ release mechanisms, *i.e*. IP_3_ and ryanodine receptors. This newly discovered ability of emetine to release Ca^2+^ from the GA may explain why chronic consumption of ipecac syrup has muscle side effects.

## Introduction

A tight control of cellular Ca^2+^ homeostasis is a typical characteristic of all eukaryotes. Cells are equipped with a complex Ca^2+^ toolkit, which enables them to maintain cytosolic Ca^2+^ concentrations ([Ca^2+^]_c_) at a very low level (~100 nM). This is achieved by the concerted action of Ca^2+^ extrusion mechanisms localized in the plasma membrane (PM), Ca^2+^ storage within the lumen of intracellular compartments, and is facilitated by Ca^2+^ buffering proteins within the cytosol and organelle lumen^1^. Changes in the [Ca^2+^]_c_ or within cellular organelles control different processes, *e.g*. excitation-contraction coupling, neurotransmission, hormone secretion, gene transcription, apoptosis and metabolism, among others^2^. However, high and prolonged increases of [Ca^2+^]_c_ are cytotoxic^3^. Regarding Ca^2+^ control within cellular organelles, the endoplasmic reticulum (ER), or its specialized version in muscle cells called the sarcoplasmic reticulum (SR), represents the most abundant storage compartment. It possesses a well characterized ATP-dependent Ca^2+^ accumulation mechanisms, the sarco/endoplasmic reticulum Ca^2+^-ATPases (SERCA), as well as two Ca^2+^ release channels; the inositol trisphosphate (IP_3_) receptors (IP_3_R) and/or the ryanodine receptors (RyR)^4,5^. SERCAs are strongly and specifically inhibited by thapsigargin and cyclopiazonic acid^6,7^. With regard to other organelles, the Ca^2+^ uptake and release mechanisms of mitochondria have been the subject of intense investigation in the last decade and their main molecular and functional characteristics have been unraveled in great detail^8,9^. In contrast, characterization of the Ca^2+^ homeostatic system of other cellular organelles, *i.e*. endosomes, lysosomes^10,11^, secretory vesicles^12^ and the Golgi apparatus (GA)^13–15^, which are characterized by a luminal acidic pH (and often collectively named “acidic Ca^2+^ stores”), is still poorly understood. For instance, a well characterized Ca^2+^ uptake mechanism in the GA is the secretory pathway Ca^2+^-ATPase (SPCA)^16,17^ and it has been shown that this Ca^2+^ pump coexists with a Ca^2+^/H^+^ exchanger^18^, which might have a minor role in accumulating Ca^2+^ given the small pH gradient between the lumen of the GA and cytosol^19^. As for Ca^2+^ release, evidence has been provided suggesting that these organelles (lysosomes in particular) possess the two-pore channel^20^ (TPC2), which is sensitive to nicotinic acid adenine dinucleotide phosphate (NAADP). Nevertheless, it should be noted that the role of TPC2 in Ca^2+^ release and its activation by NAADP has recently been challenged and the issue remains unresolved^21^.

As to the GA, the recent development of genetically encoded Ca^2+^ indicators (GECIs) selectively targeted to the GA sub-compartments has revealed a highly complex Ca^2+^ signaling toolkit in this organelle, that differs in the specialized sub-regions. The current model suggests that the cis-medial section of the GA resembles the ER in terms of its Ca^2+^ handling: Ca^2+^ release can be triggered by IP_3_ and, where RyRs are expressed, by caffeine; while Ca^2+^ uptake is catalyzed by SERCA and, in the medial-Golgi, also by SPCA^17^. The trans-Golgi region appears not to express IP_3_Rs or SERCAs and Ca^2+^ uptake involves the SPCA. In neonatal cardiomyocytes, the trans-Golgi appears to express RyRs and accordingly it releases Ca^2+^ in response to caffeine^16^. Indirect evidence suggests that the Ca^2+^ level within the GA lumen modulates vesicular trafficking and the correct sorting of proteins^22^.

While using emetine to study the well-established role of Sec61 translocon in the ER Ca^2+^ leak^23^, we found that this alkaloid, which is also present in ipecac syrup^24^, was able to reduce the Ca^2+^ content of an undefined Ca^2+^ store in HeLa cells. Ipecac syrup is an emetic agent^25^ and its chronic consumption results in reversible myopathy and cardiomyopathy^26^. At the molecular level, this alkaloid acts as an inhibitor of protein synthesis^27^, by targeting the small subunit of ribosomes^28^, without actually detaching the ribosomal complex from the translocon^29^.

Using a variety of probes and experimental approaches, here we demonstrate that emetine is capable of mobilizing Ca^2+^ from the medial- and trans-regions of the GA, without affecting the ER Ca^2+^ content. Emetine may represent the first member of a chemical library that will enable studies on the mechanism of GA Ca^2+^ homeostasis and its role in Ca^2+^ pathophysiology.

## Results

### Emetine decreases the [Ca^2+^]_L_ in an intracellular store different from the ER

**Figure 1:**
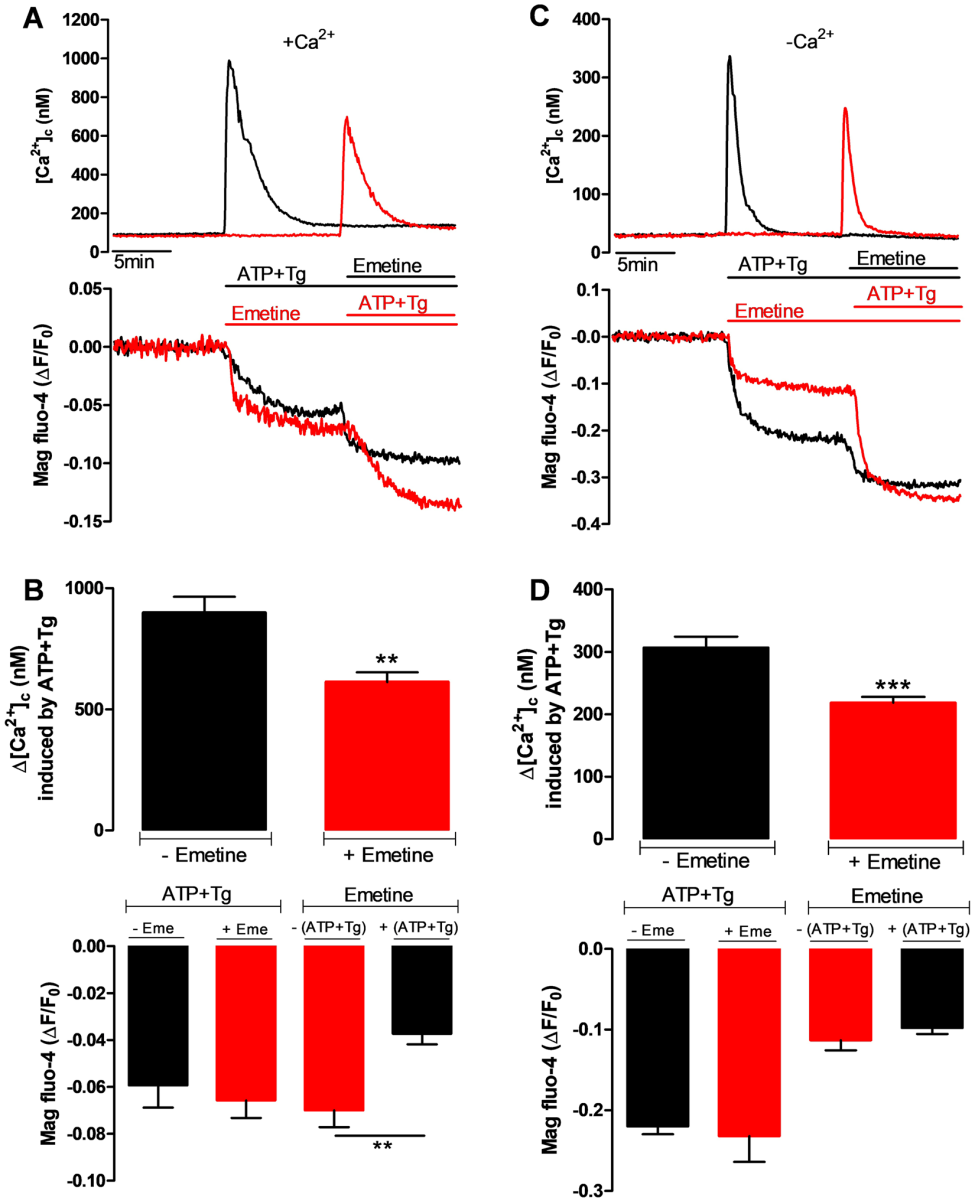
Emetine decreases [Ca^2+^]_L_ in an intracellular store different to the ER. (**A**) Representative simultaneous recording of changes in both [Ca^2+^]_c_ (upper panel) and [Ca^2+^]_L_ (lower panel) in HeLa cells (2 × 10^5^ cells/ml) in the presence of 1.8 mM [Ca^2+^] ( + Ca^2+^). Black traces show the effect of first adding the combination of 10 μM ATP and 1 μM Tg followed by 50 μM emetine at the times indicated by the horizontal lines. Red traces show the [Ca^2+^] responses observed when the order of addition was reversed. (**B**) The upper bar graph represents the average peak [Ca^2+^]_c_ response (n = 5) to the combination of ATP and Tg in the absence of emetine (black bar) or in the presence of emetine (red bar). The lower bar graph (left side) shows the maximal reduction in the [Ca^2+^]_L_ in response to ATP and Tg in the absence of emetine (black bar) or in the presence of emetine (red bar). The right side of the bottom part displays the maximal reduction in the [Ca^2+^]_L_ induced by emetine before (red bar) or after application of ATP and Tg (black bar). (**C**) Representative traces of a typical experiment, performed as described in A) except that these were carried out in the absence of external Ca^2+^ and with 0.1 mM EGTA (-Ca^2+^). Smaller agonist-induced [Ca^2+^]_c_ responses were obtained than those in the presence of external Ca^2+^. (**D**) Bar graphs represent the average peak [Ca^2+^]_c_ (top) or the reduction in the [Ca^2+^]_L_ (bottom) elicited by ATP and Tg in presence (left red bar, n = 8) or absence (left black bar, n = 10) of emetine or those in response to emetine, before or after the addition of ATP and Tg (right side ones). Data are presented as mean ± SEM and n is the number of independent experiments. Notice that emetine decreased the ATP and Tg-induced [Ca^2+^]_c_ rise without having any effect on the associated [Ca^2+^]_L_ drop regardless of the external [Ca^2+^].

To investigate the effect of emetine on Ca^2+^ homeostasis, we carried out simultaneous recordings of the signals from two fluorescent indicators; Fura-2 and Mag-Fluo-4, in HeLa cells. While Fura-2 was homogeneously distributed in the cytosol and nucleus (and thus it is a *bona fide* cytosolic and nuclear Ca^2+^ indicator, [Ca^2+^]_c_), Mag-Fluo-4 fluorescence was not distributed in a homogenous manner. Rather, a faint signal was observed in the region corresponding to the nuclear membrane, as well as reticular structures in the cytoplasmic region, while the nucleus itself was completely dark (Supplementary Fig. 1A). Vesicular structures some of which were located in the perinuclear region and colocalized with a GA marker, also displayed strong fluorescence (Supplementary Fig. 1C). The majority of the Mag-Fluo-4 fluorescence signal (75%) colocalized with both the ER and the GA (Supplementary Fig. 1B,D), therefore we consider this fluorescence signal as an indicator of luminal Ca^2+^ concentration ([Ca^2+^]_L_). In the presence of external [Ca^2+^], the combination of ATP (an IP_3_-generating agonist) and thapsigargin (Tg, an irreversible SERCA pump inhibitor) induced a transient increase in the [Ca^2+^]_c_ and this was associated with a sustained reduction in the [Ca^2+^]_L_ signal (Fig. 1A, black trace). Ten minutes later, the addition of emetine (50 μM) resulted in a further reduction in the [Ca^2+^]_L_ without any change in the [Ca^2+^]_c_. Reversing the order of addition, *i.e*., emetine first followed by ATP and Tg, resulted in an initial decrease in the [Ca^2+^]_L_, followed by a further reduction of the signal upon addition of ATP and Tg (Fig. 1A, red trace). The changes in the [Ca^2+^]_c_ were qualitatively similar using either protocol, *i.e*. no rise upon addition of emetine and a transient increase in response to ATP and Tg. However, the peak [Ca^2+^]_c_ elicited by ATP and Tg was significantly smaller when they were added after emetine (Fig. 1B, upper panel), while the reduction in the [Ca^2+^]_L_ induced by emetine was significantly larger when this alkaloid was added as the first stimulus (Fig. 1B, lower panel, second red bar).

To exclude any effect of emetine on Ca^2+^ influx across the PM (*e.g*., capacitative Ca^2+^ entry, CCE), we carried out the same experiment in the absence of external [Ca^2+^] (Fig. 1C). Qualitatively, the results were similar to those obtained in medium containing 1.8 mM CaCl_2_, however the amplitude of the [Ca^2+^]_c_ peaks were substantially reduced (Fig. 1D, upper panel) and the reductions in the [Ca^2+^]_L_ caused by ATP and Tg and by emetine were larger, suggesting that the absence of external Ca^2+^ facilitated store depletion. Similar to what was observed in presence of external Ca^2+^, the ATP and Tg-triggered [Ca^2+^]_c_ response was decreased by the presence of emetine (Fig. 1C, red trace). This effect cannot simply be explained by the cells being maintained in the absence of external Ca^2+^ for a prolonged period of time, since this 10 minute period had no effect on the amplitude of agonist-induced [Ca^2+^]_c_ response (Supplementary Fig. 2). The finding that emetine had no effect on the [Ca^2+^]_L_ responses induced by ATP and Tg and vice versa (Fig. 1D, lower panel) suggests that emetine releases Ca^2+^ from a compartment that does not contain SERCA or IP_3_ receptors, and is thus distinct from the ER.

Emetine did not increase the [Ca^2+^]_c_ at any of the concentrations tested (Supplementary Fig. S3A). However, the reduction in the [Ca^2+^]_L_ response showed a concentration dependence (Supplementary Fig. 3B) between 10 and 20 μM (Supplementary Fig. 3C). In the absence of external [Ca^2+^], the emetine-induced [Ca^2+^]_L_ response was larger and kinetically displayed two phases (Supplementary Fig. 3D, black trace). Isoemetine (50 μM), which is an isomer of emetine that does not block protein synthesis^30^, also reduced the [Ca^2+^]_L_, although its effect was slower and of smaller amplitude (Supplementary Fig. 3D, red trace).

### Emetine releases Ca^2+^ from an acidic intracellular Ca^2+^ store

**Figure 2:**
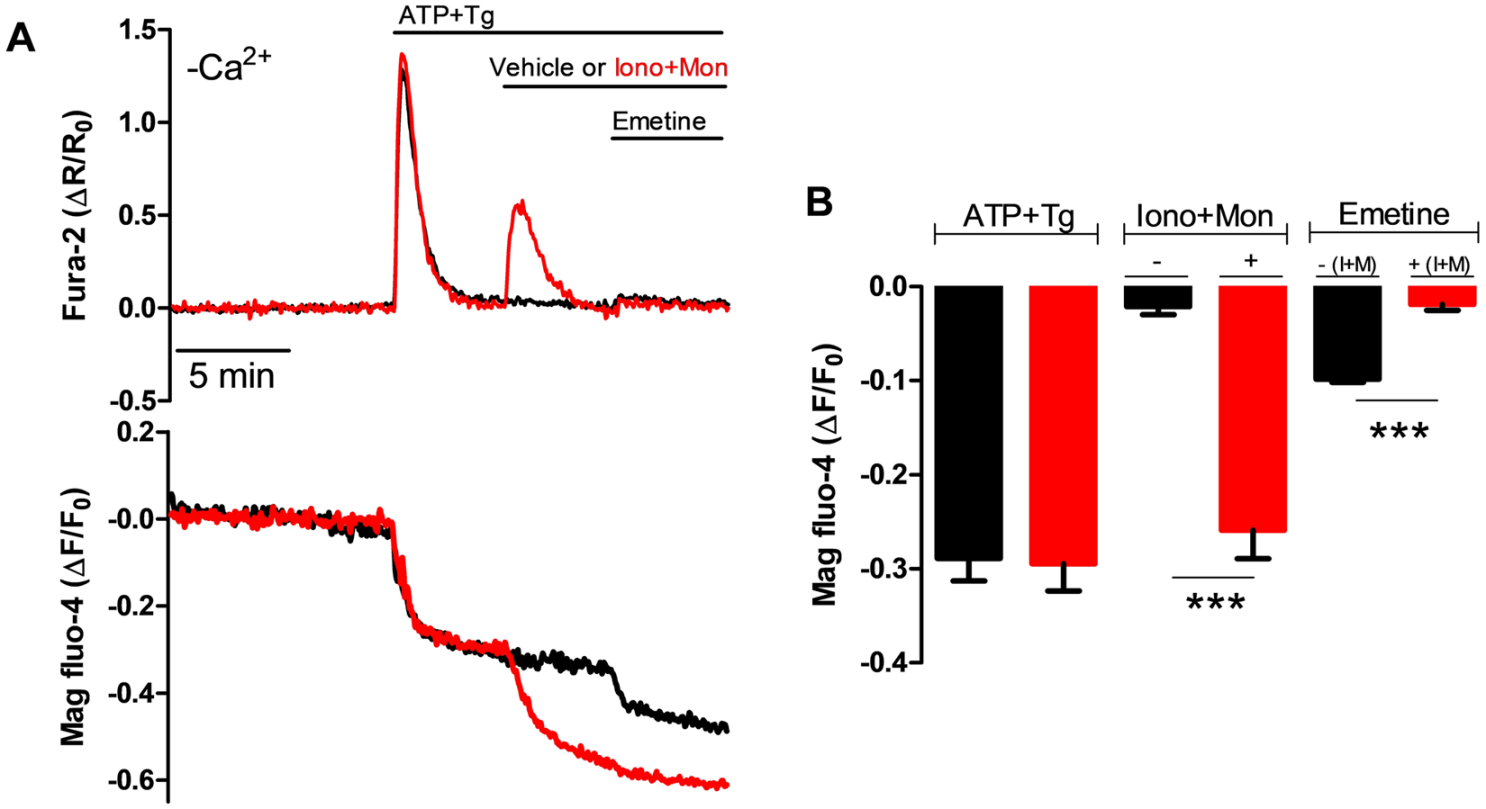
Depleting the acidic Ca^2+^ stores using the combination of ionomycin and monensin inhibited the emetine-induced [Ca^2+^]_L_ response. (**A**) Representative simultaneous recordings of changes in the [Ca^2+^]_c_ and [Ca^2+^]_L_ in HeLa cells (2 × 10^5^ cells/ml) in the absence of external Ca^2+^ and in the presence of 0.1 mM EGTA (-Ca^2+^). HeLa cells were exposed to the combination of ATP and Tg (to deplete the ER Ca^2+^ store), followed by the addition of 1 μM ionomycin (iono) and 10 μM monensin (mon), to deplete acidic Ca^2+^ stores (red traces) or vehicle alone (black traces) and then emetine was added at the time indicated. (**B**) Average reduction in the [Ca^2+^]_L_ in response to three different conditions: ATP and Tg, the combination of ionomycin and monensin (I + M), and the addition of emetine in cells where the acidic Ca^2+^ stores were either eliminated (red bars) or maintained intact (black bars). The ionophores strongly inhibited the emetine-induced [Ca^2+^]_L_ response. Data are presented as mean ± SEM of n = 3 independent experiments.

It has been demonstrated that cells contain two different types of intracellular Ca^2+^ stores, the first is represented primarily by the ER/SR and the other is characterized by having an acidic lumen, and is collectively named “acidic Ca^2+^ stores”^31^. A typical characteristic of the acidic Ca^2+^ stores is that they can be rapidly depleted by the combination of ionomycin (1 μM) and a substance that can collapse the acidic pH gradient, such as monensin (10 μM)^32^. In the experiments presented in Fig. 2, we have used the combination of ATP and Tg to deplete the ER, followed 10 min later by the combination of ionomycin and monensin. The latter treatment, resulted in a small rise in [Ca^2+^]_c_ and a large reduction in the [Ca^2+^]_L_ (Fig. 2A, lower panel). More importantly, the emetine-induced [Ca^2+^]_L_ decrease was inhibited by depletion of the acidic Ca^2+^ stores (Fig. 2B). This data suggests that emetine was targeting acidic Ca^2+^ stores to mobilize Ca^2+^.

**Figure 3:**
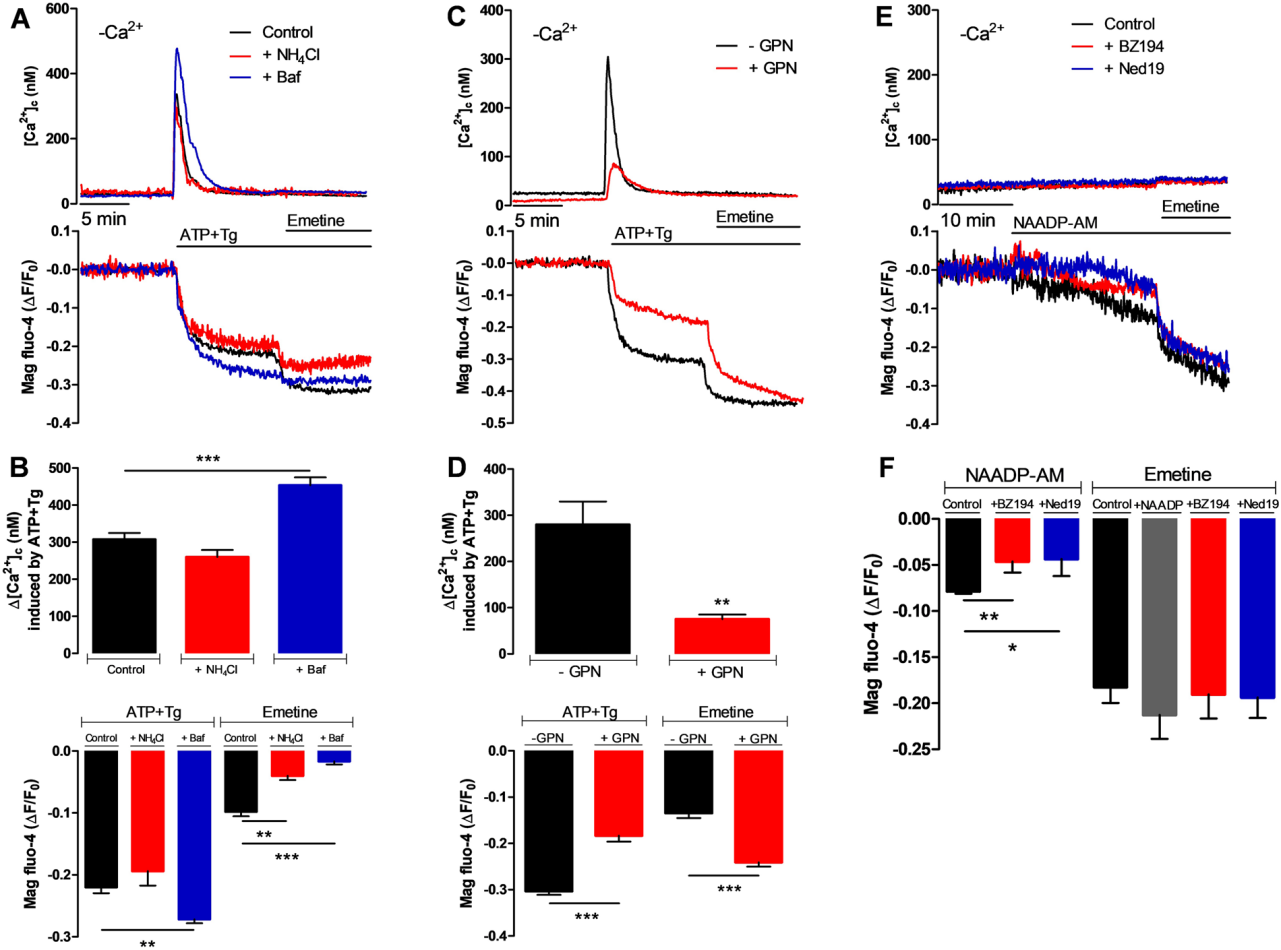
The acidic intracellular Ca^2+^ stores sensitive to emetine are not lysosomes. All experiments were carried out in the absence of external Ca^2+^ and in the presence of 0.1 mM EGTA (-Ca^2+^) in HeLa cells (2 × 10^5^ cells/ml). (**A**) Representative simultaneous recordings of changes in both [Ca^2+^]_c_ (top) and [Ca^2+^]_L_ (bottom) in cells exposed to 10 μM ATP and 1 μM Tg followed by 50 μM emetine, at the time indicated. Cells were untreated or incubated with either 18 mM NH_4_Cl or 100 nM bafilomycin before initiating recording of Ca^2+^ changes. (**B**) Bar graphs represent the average peak [Ca^2+^]_c_ response (top) elicited by ATP and Tg or the reduction in the [Ca^2+^]_L_ (bottom) elicited by ATP and Tg (left side bars) followed by emetine (right side bars) in control (black bars, n = 10), NH_4_Cl-treated cells (red bars, n = 3) or baf-treated cells (blue bars, n = 4). (**C**) Representative traces of changes in the [Ca^2+^]_c_ (top) and the [Ca^2+^]_L_ (bottom) from cells treated with 200 μM GPN (red trace) versus control cells (black trace). (**D**) Bar graphs represent the average peak [Ca^2+^]_c_ (top) or the maximal decrease of the [Ca^2+^]_L_ (bottom) elicited both by ATP plus Tg and the emetine-induced [Ca^2+^]_L_ response (right side bars) in control (black bars, n = 4) or GPN-treated cells (red bars, n = 5). (**E**) Representative simultaneous recordings of changes in both [Ca^2+^]_i_ (top) and [Ca^2+^]_L_ (bottom) in response to 10 nM NAADP-AM and followed by 50 μM emetine, added where indicated (black trace). Another set of cells were treated with NAADPR blockers BZ194 (100 μM, red trace) or Ned 19 (50 μM, blue trace) before the addition of NAADP-AM and emetine. (**F**) Bar graphs on the left side represent the average reduction in the [Ca^2+^]_L_ elicited by NAADP-AM (black, n = 8), and the inhibition of this effect in cells treated with BZ194 (red, n = 4) or Ned 19 (blue, n = 4). Bars graphs on the right side indicate the control emetine-induced reduction in the [Ca^2+^]_L_ (black bar) and the effect of treating cells with NAADP-AM (gray bar, n = 4); with NAADP-AM plus BZ149 (red bar, n = 4) and NAADP-AM plus Ned 19 (blue bar, n = 4) previous to the addition of emetine. Data are presented as mean ± SEM of n number of independent experiments.

To further corroborate that the emetine-sensitive intracellular Ca^2+^ release is from the acidic Ca^2+^ stores, the proton gradient of these compartments was collapsed using two approaches: incubation with either NH_4_Cl (18 mM) or bafilomycin (baf, 100 nM). This NH_4_Cl concentration was selected to avoid having an effect on the IP_3_-induced Ca^2+^ release, while the baf concentration was low enough to be specific for vacuolar H^+^-ATPase^33^. The rise in [Ca^2+^]_c_ and the associated drop in [Ca^2+^]_L_ induced by ATP and Tg were unaffected by pre-incubation with NH_4_Cl for 15 minutes (Fig. 3A, red trace); whereas 15 minutes pre-incubation with baf significantly increased both the [Ca^2+^]_c_ peak and the corresponding reduction in the [Ca^2+^]_L_ (Fig. 3A, blue trace). The subsequent emetine-induced decrease in [Ca^2+^]_L_ was strongly inhibited by NH_4_Cl and almost abolished by baf (Fig. 3B, lower panel). Similar results were obtained when emetine was used as the first stimulus (Supplementary Fig. 4A,B**)**. These effects can be explained by the fact that baf increases the [Ca^2+^]_L_ (Supplementary Fig. 4A, inset) as we have previously reported^34^. These results further point to the acidic Ca^2+^ stores as being the target of emetine.

To determine whether lysosomes are the target of emetine; HeLa cells were pre-incubated with the dipeptide glycyl-L-phenylalanine-2-naphthylamide (GPN). This is a substrate of the intralysosomal protease cathepsin C, and accumulation of the hydrolysis product therein will osmotically disrupt the lysosomes^35^. Surprisingly, GPN caused a dramatic inhibition of both the [Ca^2+^]_c_ rise and the [Ca^2+^]_L_ decrease induced by ATP and Tg (Fig. 3C, red trace), suggesting that this drug has a strong effect on the ER, in addition to affecting lysosomes. The emetine-induced [Ca^2+^]_L_ drop was clearly enhanced by GPN pre-treatment (Fig. 3D, lower panel), indicating that lysosomes are not the target for emetine. As shown in Supplementary Fig. 4C (black trace) the acute addition of GPN increased the [Ca^2+^]_c_ and inhibited the response to ATP and Tg, while the previous depletion of the ER with ATP and Tg abolished the effect of GPN on both [Ca^2+^]_L_ and [Ca^2+^]_c_ (Supplementary Fig. 4C, red trace). Since GPN has non-specific effects on the ER (Supplementary Fig. 4D**)**, we searched for a more specific treatment to release Ca^2+^ from the lysosomal compartment. To this end, we treated HeLa cells with NAADP/AM, a membrane permeable NAADP analogue believed to be a specific lysosomal Ca^2+^ mobilizer via TPC2 channels^36^. The application of 10 nM NAADP-AM resulted in a slow but significant drop in the [Ca^2+^]_L_ without any effect on the [Ca^2+^]_c_ (Fig. 3E, black trace). The addition of BZ194 (an inhibitor of the NAADP receptor) or Ned19 (a blocker of TPC2), inhibited the effect of NAADP-AM on the [Ca^2+^]_L_ (Fig. 3E, red and blue traces). However, none of these reagents modified the emetine-induced reduction in the [Ca^2+^]_L_ (Fig. 3F). Collectively, these data support the idea that neither lysosomes nor TPC2 channels are targeted by emetine.

### The Ca^2+^ store responding to emetine is labile and localized in the perinuclear region

**Figure 4:**
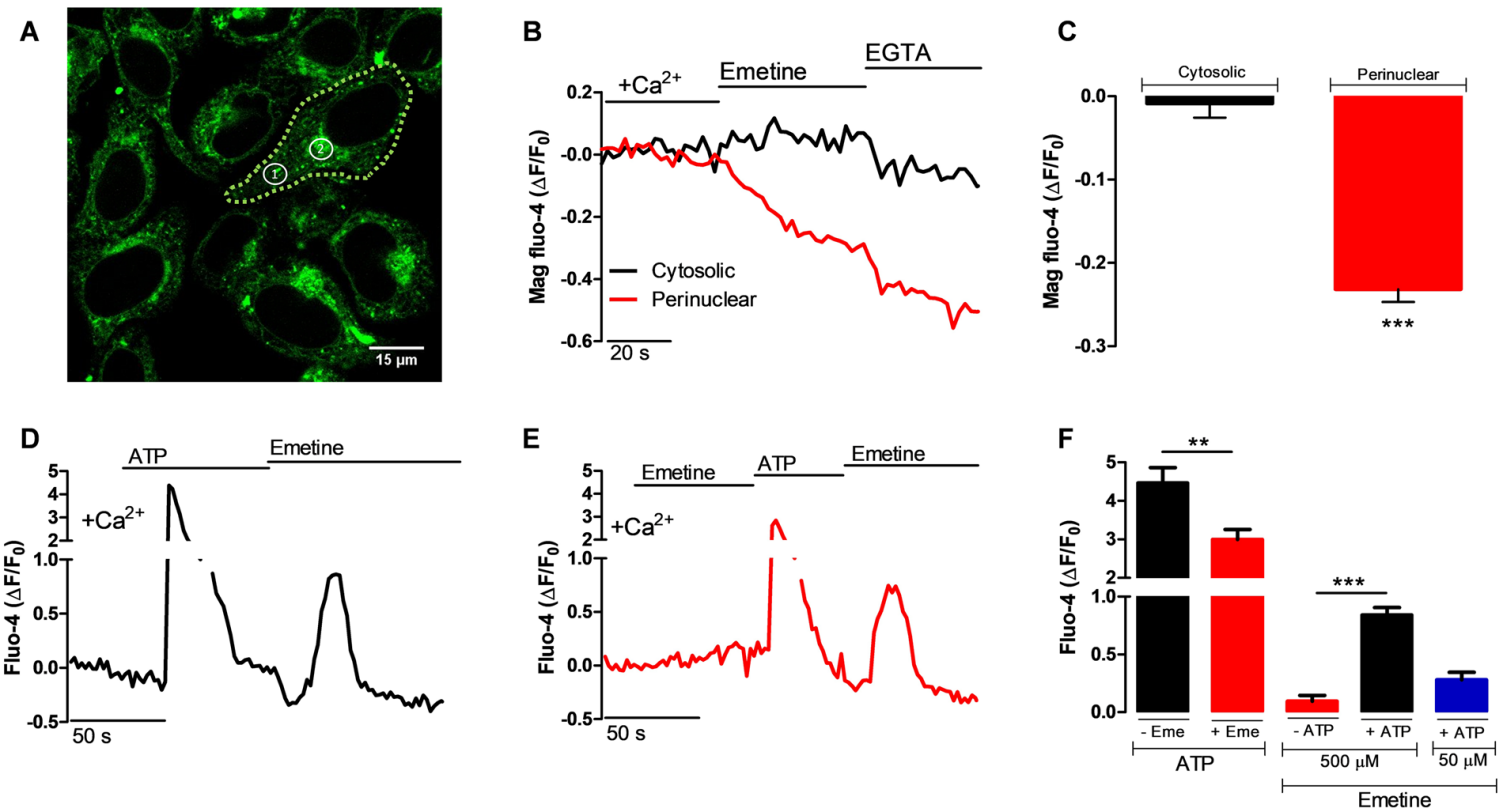
There is a perinuclear intracellular Ca^2+^ store that is labile and sensitive to emetine. All these experiments were carried out in the presence of 1.8 mM external [Ca^2+^] ( + Ca^2+^) and in single HeLa cells. (**A**) Confocal image of cells loaded with Mag-Fluo-4. The dash line delineates a cell where cytosolic (ROI 1) and perinuclear (ROI 2) regions of interest have been marked for analysis. (**B**) Representative recording showing the effect of 50 µM emetine on the Mag-Fluo-4 fluorescence in the ROIs identified in panel A). (**C**) Average maximal reduction in the Mag-Fluo-4 fluorescence in response to emetine in cytosolic (black bar) and perinuclear ROIs (red bar, n = 24 cells). (**D**) Representative recording from Fluo-4/AM-loaded HeLa cells showing the changes in [Ca^2+^]_c_ in response to the perfusion with 30 μM ATP followed by perfusion with 500 μM emetine. (**E**) In this case, emetine was perfused to naïve cells followed by ATP, and then there was a second addition of emetine as indicated. (**F**) The bar graph shows that the average peak [Ca^2+^]_c_ response to ATP was decreased after the addition of emetine (left red bar, n = 42 cells) when compared with the control (left black bar, n = 52 cells). The [Ca^2+^]_c_ response was absent when emetine was applied as the first stimulus (right red bar), but there was a significant increase in [Ca^2+^]_c_ when emetine was applied after ATP (right black bar). Similar results were obtained with 50 μM emetine but the amplitude was smaller due to the perfusion system being slow (F, blue bar). Data are presented as mean ± SEM where n indicates the number of cells studied.

To gain an insight into the identity of the Ca^2+^ store responding to emetine, we performed confocal microscopy experiments in HeLa cells loaded with Mag-Fluo-4, in the presence of external [Ca^2+^]. The perinuclear and cytosolic regions of the cells that were labelled with Mag-Fluo-4 (Fig. 4A) were then examined during the application of emetine. Addition of emetine resulted in a net decrease in the Mag-Fluo-4 fluorescence, but only in the perinuclear region; there was no significant change in the cytosolic region (Fig. 4B,C). The mean Fura-2 signals, that were obtained as shown in Figs 1–3, represent the combined response of half a million cells in suspension in a cuvette, therefore, small localized increases in cytosolic Ca^2+^ cannot be detected by this approach. To investigate whether emetine was producing a localized increase in the [Ca^2+^]_c_, cells were loaded with another cytosolic Ca^2+^ indicator, Fluo-4, and analyzed by confocal microscopy. Unlike the results obtained with Fura-2 in the cell populations, emetine was able to trigger a localized and small [Ca^2+^]_c_ response, 4-5 fold smaller than that induced by ATP (Fig. 4D). Noteworthy, this rise in perinuclear [Ca^2+^]_c_ was observed only when emetine was added within 45 seconds after ATP addition (Fig. 4E). As observed in the experiment presented in Fig. 1, pre-treatment with emetine decreased the amplitude of the ATP-induced [Ca^2+^]_c_ response (Fig. 4F). These data indicate that emetine induces a small and localized [Ca^2+^]_c_ response in the perinuclear region; but only shortly after the ER has released its Ca^2+^, suggesting that the emetine-sensitive intracellular compartment is a labile Ca^2+^ store because emetine can only increase the [Ca^2+^]_c_ in a short time window.

### Emetine does not release Ca^2+^ from the ER nor does it affect mitochondrial Ca^2+^ handling

**Figure 5:**
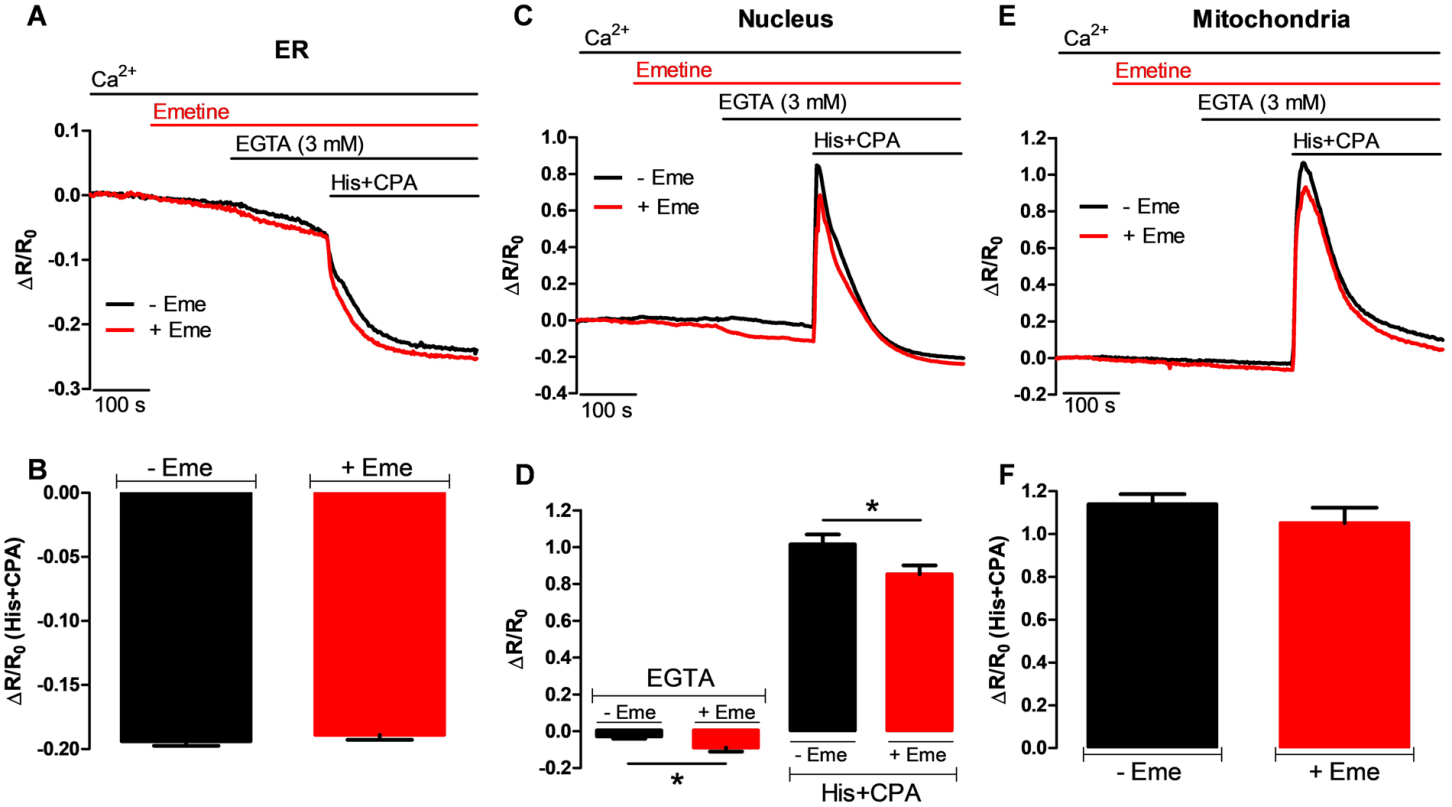
The effect of emetine on Ca^2+^ dynamics in the ER, nucleus and mitochondria, as determined with GECIs. (**A**) Average changes in ΔR/Ro for single HeLa cells that were transfected with D4ER probe and they were placed in the presence (red trace) or absence (black trace) of 50 µM emetine. Where indicated, EGTA (to chelate external Ca^2+^) and then histamine (100 µM) plus CPA (20 µM, to trigger ER Ca^2+^ release) were added. (**B**) Maximal reduction in the D4ER ΔR/ΔRo ratio in response to the combination of His and CPA with (red bar, n = 14 cells) or without (black bar, n = 15 cells) emetine. (**C**) HeLa cells were transfected with the nuclear probe H2BD3cpv. It is shown the average ΔR/Ro ratio for this probe in cells that were exposed to emetine (red trace) or vehicle (black trace) followed by EGTA and His plus CPA (to trigger nuclear Ca^2+^ rise) at the indicated times. (**D**) Emetine (red bar, n = 10 cells) significantly decreased both the resting [Ca^2+^]_n_ and the average peak [Ca^2+^]_n_ in response to agonists when compared with control cells (black bar, n = 12 cells). (**E**) Average traces of HeLa cells transfected with the mitochondrial Ca^2+^ probe, 4mtD3cpv, that were incubated with (red trace) or without (black trace) emetine. EGTA and His plus CPA (to trigger mitochondrial Ca^2+^ uptake) were added at the indicated time. (**F**) Average peak for normalized 4mtD3cpv fluorescence ratio in response to His plus CPA with (red bar, n = 23 cells) or without (black bar n = 19 cells) emetine. Data are presented as ΔR/R_0_ (see materials and methods). Data are presented as mean ± SEM where n indicates the number of cells studied.

The data presented above provide strong, but still indirect evidence that the intracellular Ca^2+^ pool sensitive to emetine is neither the ER nor lysosomes/endosomes. To directly corroborate these conclusions, we took advantage of GECIs that are specifically targeted to different organelles. In particular, we used GECIs belonging to the cameleon family, *i.e*. probes based on Ca^2+^-dependent changes in FRET (Förster/Fluorescence Resonance Energy Transfer). In the experiment presented in Fig. 5, HeLa cells were transfected with GECIs targeted to the ER (D4ER)^37^, the nucleus (H2BD3cpv)^37^ and the mitochondrial matrix (4mtD3cpv)^38^. For technical reasons, in these experiments we mainly employed His (100 µM), as the IP_3_-generating agonist and CPA (20 µM) as a reversible SERCA inhibitor. We first investigated the dynamics of ER [Ca^2+^]. As expected, His and CPA caused a very large decrease in the ΔR/R_0_ (see materials and methods, Fig. 5A) and the presence of emetine did not have any effect on His and CPA-induced reduction of the [Ca^2+^]_ER_ (Fig. 5B). As expected from the previous studies with ATP and Tg, where emetine reduced the [Ca^2+^]_c_ response, measurement of [Ca^2+^]_n_ in response to His and CPA (Fig. 5C) was clearly reduced by the application of emetine (Fig. 5D). Interestingly, in the absence of external [Ca^2+^] (EGTA), the application of emetine significantly reduced the [Ca^2+^]_n_ (Fig. 5D).

Finally, we studied HeLa cells expressing 4mtD3cpv, a mitochondrial matrix localized Ca^2+^ probe. As expected, His and CPA caused a rapid increase in mitochondrial [Ca^2+^]; however, the prior application of emetine did not affect the amplitude of the agonist-induced mitochondrial Ca^2+^ response (Fig. 5E,F).

### Emetine releases Ca^2+^ from the Golgi apparatus

The experiments described so far, which have been carried out using both chemical Ca^2+^ indicators and GECIs, suggested that the GA might be the main target for emetine. The GA is an organelle preferentially localized in the perinuclear region^39^, its luminal [Ca^2+^] is high^16^ and its lumen is slightly acidic^19^. It needs to be stressed that it has been shown previously that the GA sub-compartments have quite distinct Ca^2+^ uptake and release characteristics and accordingly we have used two different GECIs targeted to the medial (medial-GoD1cpv)^17^ and the trans compartments (GoD1cpv)^16^ of the GA.

**Figure 6:**
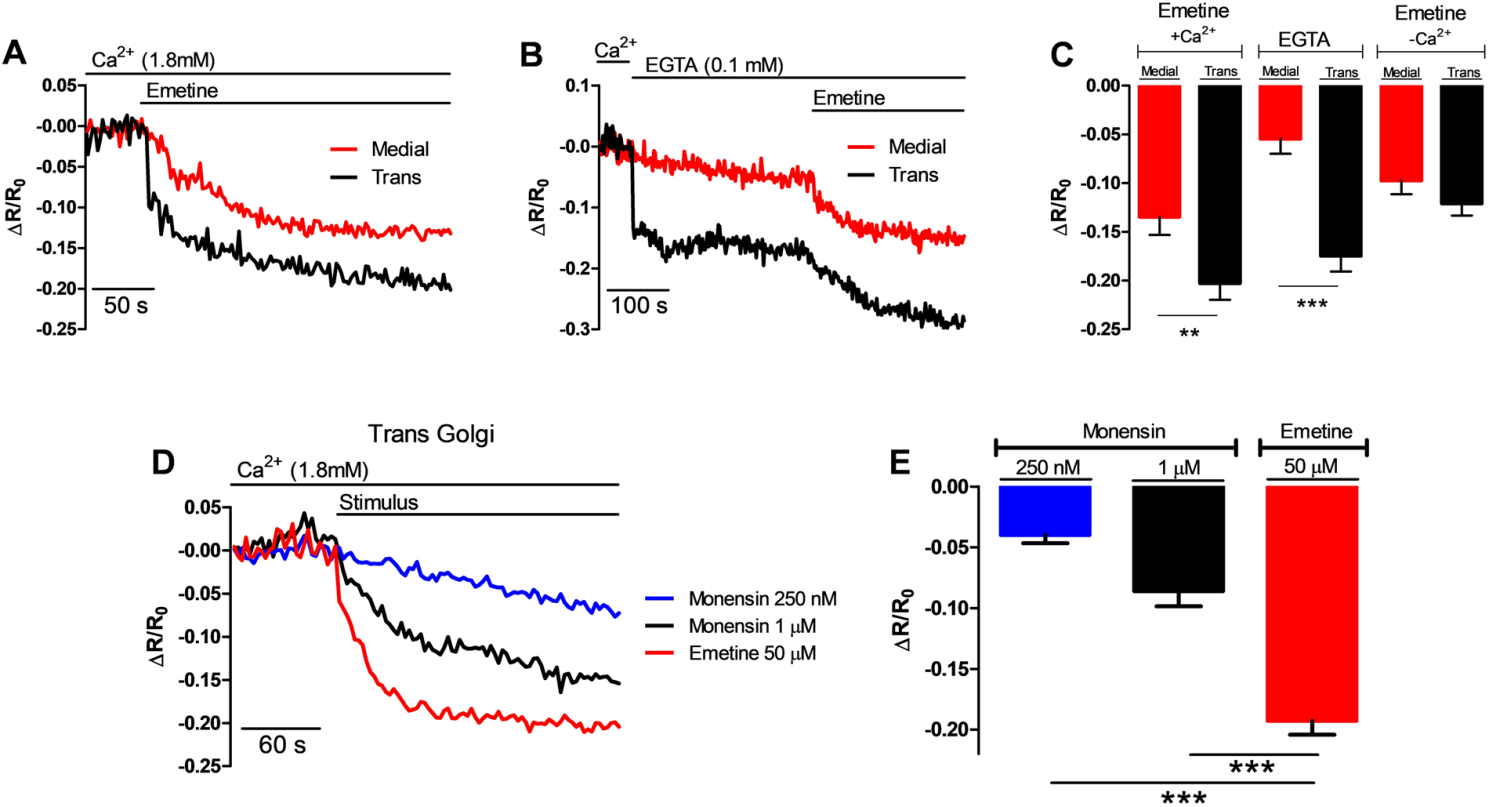
Emetine decreases the luminal [Ca^2+^] in the Golgi apparatus. (**A**) Average traces of single HeLa cells that had been transfected with either the trans-GA (GoD1cpv, black trace) or medial (medial-GoD1cpv, red trace) luminal Ca^2+^ indicators, and then exposed to emetine in the presence of [Ca^2+^]_e_ at the indicated times. (**B**) The same experiment as in panel A) except that external [Ca^2+^] was decreased by switching to Ca^2+^-free saline solution supplemented with 0.1 mM EGTA, where indicated, and this was then followed by the addition of emetine. (**C**) Emetine significantly decreased the [Ca^2+^]_L_ in the medial region (left red bar, n = 12 cells) and in the trans region (left black bar, n = 23 cells) in the presence of 1.8 mM [Ca^2+^]. The absence of external [Ca^2+^] immediately decreased the [Ca^2+^]_L_ in the trans region (middle black bar, n = 19 cells) more than in the medial region (middle red bar, n = 14 cells), while the application of emetine in the absence of external [Ca^2+^] decreased [Ca^2+^]_L_ to similar extents in both the trans (right black bar) and medial regions (right red bar). (**D**) Cells expressing the trans-GA probe were exposed, where indicated (stimulus) to either 250 nM monensin (n = 18 cells), 1 μM monensin (n = 28 cells) or 50 μM emetine (n = 32 cells). E) Emetine induced a much larger reduction in the trans-Golgi [Ca^2+^]_L_ than monensin at 90 sec. Data are presented as ΔR/R_0_. Data are presented as mean ± SEM where n indicates the number of cells studied.

Application of emetine to cells transfected with GA probes caused a strong reduction in the ΔR/R_0_ in both the medial- (Fig. 6A, red trace) and the trans-Golgi region (black trace). Unlike the ER (Fig. 5A), [Ca^2+^]_L_ in the trans-Golgi was more sensitive to reducing the external [Ca^2+^] with EGTA (Fig. 6B, black trace), in this condition, the application of emetine resulted in significant reductions in the [Ca^2+^]_L_ of both medial- and trans-Golgi regions (Fig. 6C). The fast reduction in the [Ca^2+^]_L_ in the trans-Golgi in response to the reduction of the external [Ca^2+^] adds to the notion that this GA region is a labile Ca^2+^ store.

A potential artifact with the GA targeted cameleons is that alkalization of the lumen will mimic a drop in the [Ca^2+^]^16^. To address whether emetine affects the luminal pH in the GA, we have used the same protocol as Lissandron *et al*. to investigate the pH changes in the trans-Golgi^16^. Specifically, we directly excited the acceptor of the cameleon at 510 nm and recorded the fluorescence changes at 540 nm. Under this situation, any change in the acceptor fluorescence signal is independent of the Ca^2+^ level and is due to changes in the luminal pH^16^. Supplementary Fig. 5A shows that treatment with 1 μM monensin (black trace), caused a large increase in the acceptor fluorescence; while the addition of 50 μM emetine also elevated the acceptor fluorescence (red trace), but only by about 1/3 of that induced by 1 μM monensin. The effect of emetine was similar to that induced by 250 nM monensin (blue trace). Importantly, the application of emetine resulted in a reduction in both the GA [Ca^2+^] and an increase in the luminal pH (observed as parallel fluorescence increase in the FRET donor and acceptor) in the medial-Golgi (Supplementary Fig. 5B) and trans-Golgi **(**Supplementary Fig. 5C). The application of EGTA, to reduce external [Ca^2+^], resulted in antiparallel fluorescence changes in the trans-Golgi (Supplementary Fig. 5D) confirming that this action has changed the [Ca^2+^] without altering the pH. However, although the effect of emetine on the trans-GA region pH is similar to 250 nM monensin, the changes in the ΔR/R_0_ induced by emetine were faster and larger than those induced by 250 nM monensin (Fig. 6D). Even higher concentrations of monensin (1 μM) had a smaller effect on the ΔR/R_0_ than emetine (Fig. 6E). Taken together, the data shown in Fig. 6 and Supplementary Fig. 5 demonstrate that emetine specifically targets the GA and that this alkaloid has the dual effect of mobilizing Ca^2+^ and increasing the luminal pH.

### Emetine releases Ca^2+^ from the trans-Golgi of heart HL-1 cells

**Figure 7:**
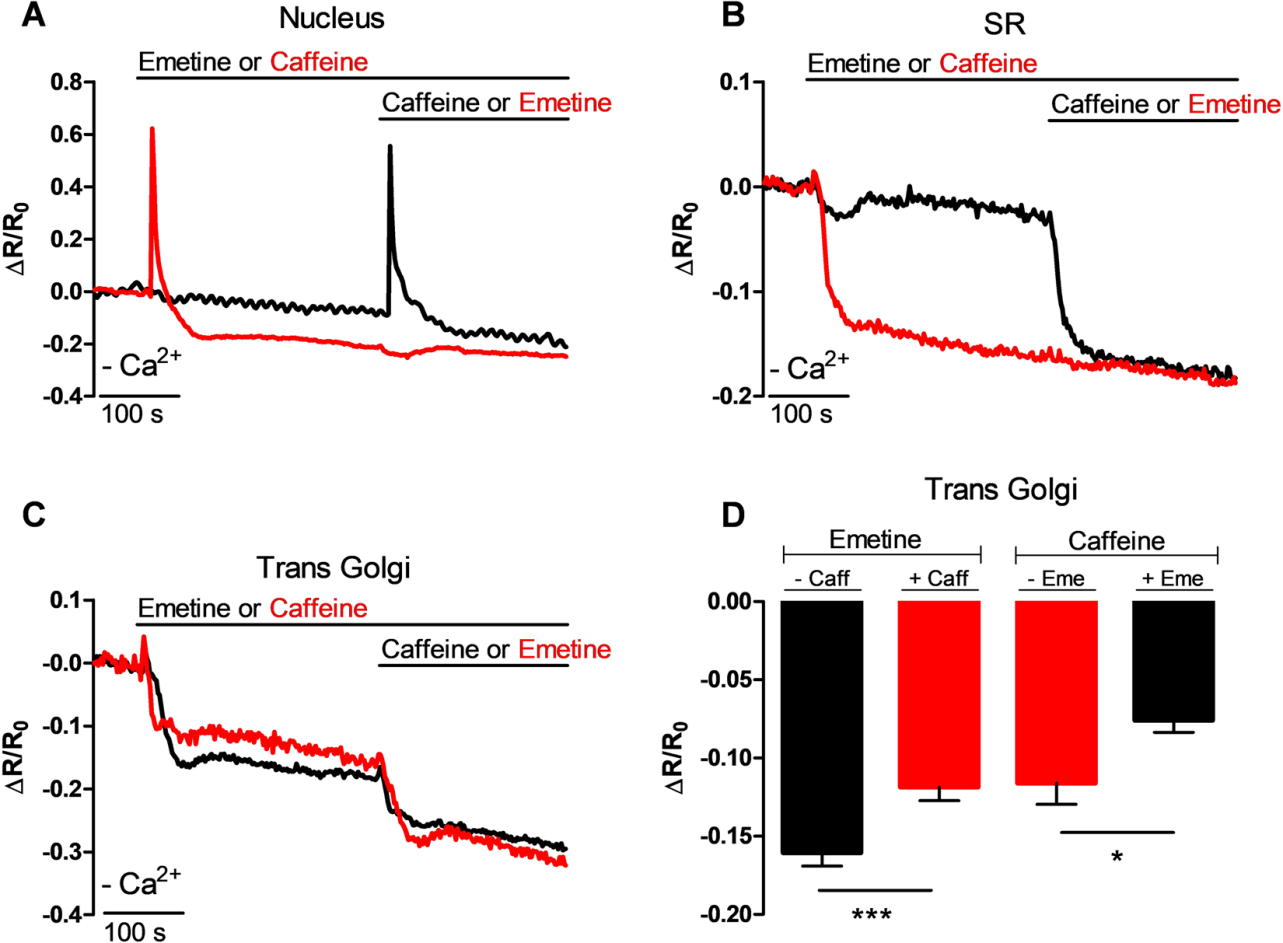
The trans-Golgi of HL-1 mouse cardiac cells is sensitive to emetine. Mouse HL-1 heart cells were transfected with H2BD3cpv (**A**), D4ER (**B**) and GoD1cpv (**C**) and maintained in the absence of external Ca^2+^ (-Ca^2+^). Changes in the average ΔR/Ro ratio for cells that were stimulated with caffeine and then emetine (red trace), whereas in other cells the application order was reversed (black trace). (**D**) Average reduction in the trans-Golgi [Ca^2+^]_L_ in response to emetine (left black bar) was significantly reduced by the previous addition of caffeine (left red bar) while emetine (right black bar, n = 14 cells) inhibited the caffeine-induced reduction in the [Ca^2+^]_L_ (right red bar, n = 17 cells) when measured at 30 seconds after the application of each substance. Data are presented as mean ± SEM where n indicates the number of cells studied.

Since the main side effect associated with chronic emetine consumption is reversible myopathy and cardiomyopathy^26^, we wondered whether emetine specifically targets the RyR (the main receptor expressed in the SR of cardiac and muscle cells). To this end, we employed HL-1 cells, a cell line derived from mouse atrial myocytes, which express RyR2^40^. Fig. 7A demonstrates that addition of caffeine, an agonist of RyRs, to HL-1 cells transfected with H2BD3cpv resulted in a transient increase in the [Ca^2+^]_n_. The application of emetine did not have any effect on the [Ca^2+^]_n_ when perfused either before or after caffeine (Fig. 7A). Furthermore, caffeine caused a decrease in the SR [Ca^2+^] of cells transfected with the SR targeted cameleon, D4ER, whereas emetine was totally without effect on the SR [Ca^2+^] either when applied before (Fig. 7B, black trace) or after caffeine (Fig. 7B, red trace). In HL-1 cells that were transfected with the trans-GA Ca^2+^ probe (GoD1cpv), emetine caused a significant reduction in the ΔR/R_0_ (Fig. 7C, black trace), and the amplitude was similar to that obtained with caffeine (Fig. 7D). Interestingly, the prior application of emetine or caffeine resulted in a slightly smaller luminal response to the other alkaloid when it was subsequently applied (Fig. 7C,D). The lack of effect of emetine on SR [Ca^2+^], where RyR2s are present, and the clear drop in the trans-Golgi [Ca^2+^] induced by emetine suggest that RyR2s might not be the target of this alkaloid. Additionally, emetine decreased the trans-GA [Ca^2+^] in two different cell types suggesting that this effect is not cell specific.

## Discussion

Emetine, a well-known inhibitor of protein synthesis^27^ also blocks the Ca^2+^ leak from the ER via translocon^23^. While we were studying the nature of this ER Ca^2+^ leak, we discovered that emetine was able to decrease [Ca^2+^]_L_ in Mag-Fluo-4 loaded HeLa cells. Our colocalization data indicated that the majority of Mag-Fluo-4 fluorescence signal comes from both the ER and the GA and this situation allowed us to discover that emetine was specifically mobilizing Ca^2+^ from the GA and not from the ER.

However, three different Ca^2+^ indicators, Fura-2, Fluo-4 and H2BD3cpv, did not show any increase in the [Ca^2+^]_c_ in response to emetine, when it was applied as the first stimulus. Interestingly, we found that releasing Ca^2+^ from the ER, appears to have loaded with Ca^2+^ the emetine-sensitive store to the extent that the application of this alkaloid produced a small and transient increase in the [Ca^2+^]_c_, which was localized only to the perinuclear region of HeLa cells. Indeed, it has been previously shown that His and CPA transiently increases the trans-GA [Ca^2+^]_L_^16^. Furthermore, the emetine-sensitive store can be considered a labile Ca^2+^ pool because the Ca^2+^ captured from the ER is lost very rapidly. In this regard, FRET experiments showed that the removal of external [Ca^2+^] decreased trans-Golgi [Ca^2+^]_L_ more rapidly than the [Ca^2+^]_L_ in the ER, supporting the idea that trans-Golgi region is a labile or leaky Ca^2+^ store that needs a constant supply of Ca^2+^ from either the ER or the external medium.

Another critical observation was that emetine reduced the amplitude of the agonist-induced [Ca^2+^]_c_ rise in HeLa cells. Since emetine neither decreased the Ca^2+^ content of the ER nor inhibited Ca^2+^ release induced by an agonist, then it is unlikely that emetine is inhibiting the IP_3_Rs or reducing the Ca^2+^ content of the ER. Additionally, decreasing the external [Ca^2+^] after application of emetine resulted in a clear reduction of the resting [Ca^2+^]_n_ as observed with H2BD3cpv. However, the molecular mechanism behind this effect of emetine is still undefined. We do not think that emetine is increasing plasma membrane-mediated Ca^2+^ extrusion, because the time course of the reduction in the agonist-induced [Ca^2+^]_i_ response was not altered by the presence of this alkaloid. Moreover, emetine did not decrease the amplitude of the caffeine-induced [Ca^2+^]_n_ response in HL-1 cells, suggesting that the effect of emetine on reducing cytosolic Ca^2+^ responses is not generalized.

We have observed that the reduction in the [Ca^2+^]_L_ induced by the combination of ATP and Tg was smaller in the presence than in the absence of external [Ca^2+^]. We think that this difference resulted from a strong Ca^2+^-dependent inactivation of the IP_3_R, a condition that has been previously reported^41^. Nevertheless, we consider that this combination produced a functional depletion of the IP_3_R-sensitive ER Ca^2+^ store. In the presence of external [Ca^2+^], the [Ca^2+^]_L_ response to emetine was decreased only when added after the combination of ATP and Tg. This situation could be explained by the activation of CCE upon stimulation with ATP and Tg and this Ca^2+^ entry might be captured by the GA, counteracting the Ca^2+^ mobilization activity of emetine. Indeed, the [Ca^2+^]_L_ in the trans-GA region was extremely sensitive to the external [Ca^2+^], as discussed above. In the absence of external [Ca^2+^] there will be no CCE and accordingly, the [Ca^2+^]_L_ response to emetine was not modified by the previous application of ATP and Tg.

Three different approaches, and two different cell types, support the idea that emetine is not releasing Ca^2+^ from the ER or the SR. In the absence of external [Ca^2+^], emetine did not modify the depletion of the agonist-sensitive ER (ATP or histamine) in combination with SERCA pump inhibitors (Tg or CPA). Confocal experiments showed that emetine released Ca^2+^ only from the perinuclear region, where 60% of the Golgi marker colocalized with Mag-Fluo-4; a completely different picture was observed for the ER, which is distributed throughout the entire cytosol.

We decided then to study whether acidic Ca^2+^ stores were targeted by emetine, with the knowledge that Ca^2+^ handling in the acidic compartment is dependent on the proton gradient generated across this membrane^31^. We studied the role of the acidic Ca^2+^ stores with the combination of ionomycin and monensin. These ionophores produced a smaller and transient [Ca^2+^]_c_ response compared to the one produced by ATP and Tg, and yet caused a similar reduction in the [Ca^2+^]_L_. In addition, these ionophores inhibited the emetine-induced reduction in the [Ca^2+^]_L_. To further support the idea that emetine was targeting an acidic Ca^2+^ store we employed different approaches to disrupt the proton gradient of these stores, *i.e*. alkalization with NH_4_Cl^42^ and inhibition of the V-type H^+^−ATPase with bafilomycin^43,44^. This inhibitor increased the agonist-induced Ca^2+^ release from the ER, likely by decreasing the Ca^2+^ buffering activity of the acidic intracellular stores, in agreement with previous reports^45^; while NH_4_Cl did not show this effect because cellular alkalization inhibits ER Ca^2+^ release by decreasing the activity of SERCA pump^46^. We found that although bafilomycin and NH_4_Cl did not have the same effect on agonist induced Ca^2+^ release, they both decreased the emetine-induced [Ca^2+^]_L_ response by increasing the pH in the intracellular acidic stores. Since these conditions do not deplete acidic Ca^2+^ stores within the time frame used here, we think that emetine needs an acidic environment to activate the Ca^2+^ mobilization mechanism. Thus, we concluded that an acidic Ca^2+^ store was the target of emetine.

The involvement of the endo-lysosomal system was discarded because NAADP or inhibitors of the NAADP receptor did not affect the emetine-induced [Ca^2+^]_L_ response. Additionally, GPN, a lysosomal disruptor^47^, did not decrease the [Ca^2+^]_L_ response induced by emetine. Similar to previous reports^48^, we have observed that the effect of GPN on the [Ca^2+^]_c_ and the [Ca^2+^]_L_ is due to Ca^2+^ release from the ER. Indeed, complete depletion of the ER Ca^2+^ store with the combination of ATP and Tg fully abolished the effect of GPN on both the [Ca^2+^]_c_ and the [Ca^2+^]_L_. These data mean that even when GPN is targeting lysosomes, its effect on [Ca^2+^]_c_ and [Ca^2+^]_L_ somehow reflects an action on the ER.

To determine whether the GA was the target for emetine, we transfected cells with either medial-^17^ or trans-Golgi^16^ cameleons as previously reported. Both compartments responded to emetine application with a clear reduction in the [Ca^2+^]_L_. Moreover, emetine still decreased the [Ca^2+^]_L_ in these two GA compartments when cells were maintained in Ca^2+^ free extracellular medium containing EGTA, implying that emetine Ca^2+^ mobilization activity might involve the activation of an ion channel instead of blocking the Ca^2+^ loading mechanism. Since the trans-Golgi region is a labile Ca^2+^ store, this might explain why emetine does not produce any increase in the [Ca^2+^]_c_ as the first stimulus, but only right after Ca^2+^ has been released from the ER by an agonist. We have observed that emetine increases the luminal pH as well and to assess the role of pH in the effect of emetine, we titrated pH changes with monensin. These data indicate that only a small part of the effect of emetine on the reduction of the ΔR/R_0_ can be explained by its associated increase in the luminal pH, the main effect of emetine is on the reduction in the [Ca^2+^]_L_.

The effect of emetine was not limited to the GA of HeLa cells. Indeed, similar results have been obtained studying the trans-Golgi region of HL-1 atrial cells, which express RyR2^43^. The trans-Golgi region of HL-1 cells responded to both caffeine and emetine with a similar reduction in the [Ca^2+^]_L_. Interestingly, the trans-Golgi Ca^2+^ store sensitive to emetine partially overlaps with the region released by caffeine in HL-1 cells. It seems unlikely that the channel activated by emetine is the RyR2.

The SPCA is a Tg-insensitive Ca^2+^ pump responsible for Ca^2+^ uptake by the trans-Golgi^16^ whereas it co-exists with SERCA in the cis/medial-GA^17^. The fundamental importance of SPCA in the physiology of GA has been shown by a number of studies, which have reported that reduction or ablation of SPCA causes morphological alterations in the Golgi structure, as well as the disruption and dysfunction of both vesicle trafficking and protein sorting in the secretory pathway^49–51^. However, SPCA has been reported to be expressed in other secretory vesicles, where it is required for Ca^2+^ homeostasis in these organelles^52,53^. Our data demonstrate that emetine is directly and specifically affecting GA. However, it is unlikely that emetine is inhibiting SPCA; but it appears to be activing a leak channel in the GA. Further experiments are needed to unveil the molecular nature behind this effect of emetine.

This new role of emetine, as a specific Ca^2+^ mobilization agent from GA, might explain the symptoms of cardiac and skeletal myopathies observed in people who are chronically consuming emetine^24,26^. This might lead to a dysfunction in Ca^2+^ dynamics in the GA that could result in altered Ca^2+^ handling in muscle cells.

## Methods

### Reagents

Adenosine triphosphate (ATP), thapsigargin (Tg), emetine, bafilomycin A_1_ (baf), histamine (His) and cyclopiazonic acid (CPA) were purchased from Sigma-Aldrich, Glycyl-L-phenylalanine-2-naphthylamide (GPN) from Santa Cruz Biotechnology, NH_4_Cl from Merck and Ned 19-Trans from Enzo. The acetoxymethyl (AM) ester forms of organic Ca^2+^ indicators Mag-Fluo-4, Fura-2, Fluo-4 were purchased from Invitrogen (Molecular Probes). NAADP-AM and BZ194 were kindly provided by Dr. Claudia Treviño from The Biotechnology Institute of UNAM (Cuernavaca, Morelos, Mexico)^54^. Emetine was dissolved in water and hydrophobic chemicals in dehydrated dimethyl sulfoxide (DMSO) in stock 1000-fold higher than the final concentration used.

### Cell culture and transfection

HeLa cells were purchased from ATCC and cultured in Dulbecco’s modified Eagle’s medium with high D-glucose (4500 mg/l), L-glutamine and sodium pyruvate (110 mg/l) supplemented with 10% fetal bovine serum, and 100 units/mL penicillin and 100 µg/mL streptomycin at 37 °C and 5% of CO_2_ in humid conditions. For the culture of HL-1 cells, Claycomb medium (from Sigma-Aldrich) supplemented with norepinephrine (100 µM), L-glutamine (2 mM) and the same concentration of fetal bovine serum and antibiotic as mentioned above was used.

Transfection of genetically encoded Ca^2+^ indicators was carried out as follows: HeLa and HL1 cells were seeded onto 18 mm diameter coverslips (coated with 25 µg/mL fibronectin in 0.2% of gelatin for the HL1 cells) and transfected at 60% confluence with 1 µg DNA employing TransIT^®^-LT1 transfection reagent (Mirus) for HeLa cells or Lipofectamine^®^ 2000 for HL1 cells. Experiments were performed 24 h or 48 h after transfection.

### Simultaneous recordings of cytosolic and luminal [Ca^2+^] in HeLa cell population

HeLa cells were trypsinized after reaching 80% confluence. The cell suspension was microfuged and the pellet was suspended in extracellular-like saline solution containing in mM: 121 NaCl, 5.4 KCl, 0.8 MgCl_2_, 1.8 CaCl_2_, 6 NaHCO_3_, 25 HEPES and 5.5 glucose [pH 7.3 at room temperature (RT)]. Cell viability was always higher than 95% as determined by trypan blue exclusion assay and 1 × 10^6^ cells/ml were loaded with 1 µM of each of the Ca^2+^ indicators, *i.e*. Fura-2/AM and Mag-Fluo-4/AM, to determine changes in cytosolic ([Ca^2+^]_c_) and luminal ([Ca^2+^]_L_) calcium concentrations, respectively. This loading was carried out in the dark and at RT for 2 h. At the end of this time, half a million cells were microfuged twice and the cell pellet was re-suspended in 2.5 ml of saline solution with or without CaCl_2_ plus 0.1 mM EGTA, as indicated. Fluorescence signals were recorded at a dwell time of 100 ms and a frequency of 2.7 Hz with excitation wavelengths of 340, 360 and 380 nm for Fura-2 and 495 nm for Mag-Fluo-4 with the same emission wavelength of 515 nm using a DeltaRAM V PTI spectrofluorometer.

The [Ca^2+^]_c_ calculated from the Fura-2 signals and normalization of the Mag-Fluo-4 signals was carried out as previously described^55^. To discard the participation of lysosomes, cells were incubated with GPN for a time period of 10 min. Since the released naphthylamine (a product of GPN cleavage mediated by cathepsin C) interferes with Fura-2 fluorescence, cells were washed before recording. In the situation where GPN was present, we did not use the 340/380 ratio; only the 380 nm fluorescence signal was used to reflect changes in the [Ca^2+^]_c_.

The use of ionomycin and monensin to fully deplete the acidic Ca^2+^ stores interfered with the use of digitonin for calibration, as revealed by changes in the Fura-2 Ca^2+^-insensitive fluorescence at 360 nm, precluding the transformation of the 340/380 fluorescence ratio to [Ca^2+^]_c_. Thus, we have normalized the 340/380 ratio using the value at time zero (ΔR/R_0_).

### Confocal microscopy experiments

HeLa cells were cultured onto 21 × 21 mm coverslips until they reached 80% confluence. They were then loaded with 1 µM of either Mag-Fluo-4/AM or Fluo-4/AM in the dark at RT for 2 and 1 hour, respectively. After this time, the coverslip was placed onto the chamber and bathed in extracellular-like saline (composition described above). Fluorescence recordings were obtained using a Carl Zeiss LSM700 confocal inverted microscope with a 63x oil immersion objective (1.4 N.A.). Both Ca^2+^ indicators were excited with 488 nm laser line using a pinhole of 45 µm, and fluorescence images were collected every 1.93 s.

Analysis of the Ca^2+^ indicator fluorescence signals was carried out with ImageJ (Wayne Rasband, Bethesda, USA). Regions of Interest (ROIs) were drawn in two different regions: perinuclear and cytosolic. Since the Mag-Fluo-4 signal exhibited a constant exponential decrease, the first 20 images (i.e. before the perfusion of any stimulus) were fitted to this equation Y = A_0_exp^−x/t^ + y_0_ and this was considered as the basal fluorescence (F_0_). Changes in the [Ca^2+^]_L_ were calculated as ∆F/F_0._

Colocalization studies of Mag-Fluo-4 and organelles, either the ER or the GA were carried out by transfecting HeLa cells with 2 µg (4 μL of Lipofectamine 2000) of either mCh-Sec61 beta (gift from Gia Voeltz, Addgene plasmid # 49155) or mCherry-Golgi-7 (gift from Michael Davidson, Addgene plasmid # 55052). Transfected cells were also loaded with Mag-Fluo-4/AM, as described above. Live HeLa cell imaging was carried out with the confocal microscope to visualize the degree of colocalization between Mag-Fluo-4 and the probe for either the ER or the GA. The level of colocalization was quantified using Manders´ coefficient.

### FRET experiments for [Ca^2+^] determination using GECIs

FRET experiments were performed as described by Drago *et al*.^56^. Briefly, cells seeded on coverslips were placed onto an open-topped chamber at 37 °C with modified Krebs-Ringer buffer (mKRB) containing (mM): 135 NaCl, 5 KCl, 10 glucose, 1 Mg_2_Cl, 1.8 CaCl_2_ and 10 HEPES (pH 7.4 at 37 °C). Image exposure time was 200 ms and acquisition frequency 0.5 Hz. With these probes, the ratio (R) between the fluorescence intensity emitted by the acceptor (at 540 nm) and by the donor (at 480 nm) fluorophores (upon excitation of the donor) is a function of the FRET efficiency and accordingly of the [Ca^2+^]. An increase or a decrease in the 540/480 emitted fluorescence ratio thus indicates an increase, or a decrease, in the [Ca^2+^], respectively. Data are presented as a ∆R/R_0_, where: ∆R is the change of the cpV/CFP emission intensity ratio at any time, and R_0_ is the fluorescence emission ratio at the time 0.

### Statistical Analysis

All the data represent at least three independent experiments. Figures were prepared by GraphPad Prism version 5.0. Averages are expressed as mean ± SEM (n, number of independent experiments) analyzed by unpaired Student´s t test, where *P < 0.05, **P < 0.01 and ***P < 0.001 are statistically significant.

## Electronic supplementary material

Supplementary file

## Data Availability

All data generated or analyzed during this study are included in this published article (and its Supplementary Information files).
