# Supplementary material for: The Trans Golgi Region is a Labile Intracellular Ca2+ Store Sensitive to Emetine
Source: Sci Rep. 2018 Nov 21;8:17143. doi: 10.1038/s41598-018-35280-z (PMC6249204; doi:10.1038/s41598-018-35280-z)
Supplement: Supplementary file 1 — Supplementary file [file 41598_2018_35280_MOESM1_ESM.pdf]

Supplementary data for work titled

THE TRANS GOLGI REGION IS A LABILE INTRACELLULAR Ca^{2+} STORE SENSITIVE TO EMETINE

Martín-Leonardo Gallegos-Gómez[1], Elisa Greotti[2,3], María-Cristina López-Méndez[1], Víctor-Hugo Sánchez-Vazquez[1], Juan-Manuel Arias[4] and Agustín Guerrero-Hernández[1]*

[1]Department of Biochemistry, Cinvestav, Mexico City 07000, Mexico. [2]Department of Biomedical Sciences, University of Padua, Padua 35121, Italy. [3]Neuroscience Institute, Padova Section, National Research Council, Padua 35121, Italy. [4]Programa de Neurociencias-UIICSE, Facultad de Estudios Superiores Iztacala, UNAM, Av. de los Barrios 1, Los Reyes Iztacala, 54090 Estado de México, Mexico

*Correspondence should be addressed to A. G-H. (aguerrero@cinvestav.mx)

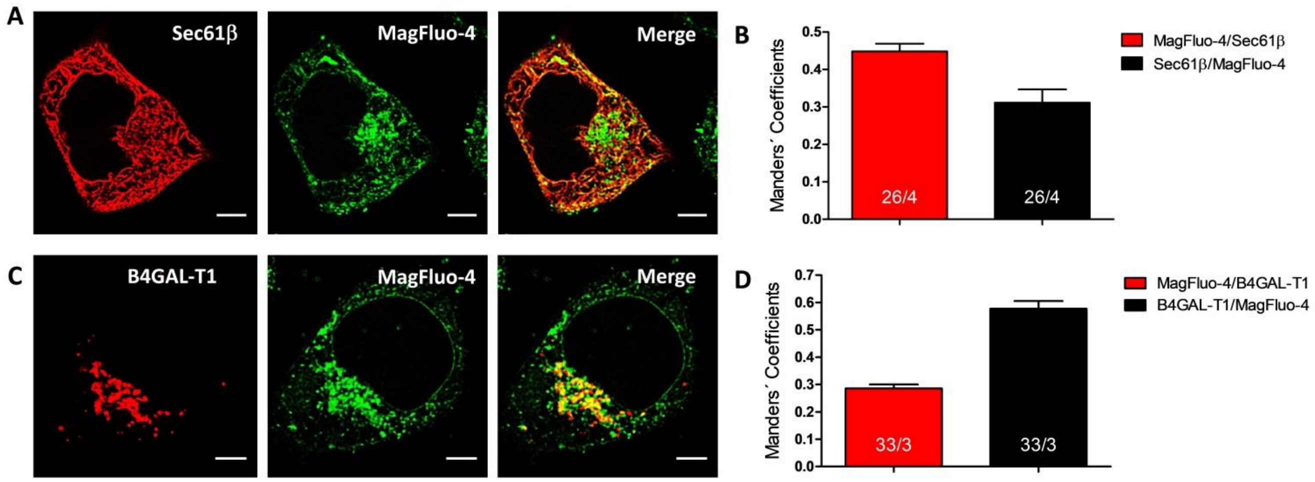

Supplementary Fig. 1 Imaging living HeLa cells transfected with markers for either the ER or Golgi apparatus and loaded with Mag-Fluo-4/AM. A) Representative image of cell transfected with mCherry-Sec61β to mark the ER and loaded with Mag-Fluo-4/AM to show luminal [Ca^{2+}], notice that the latter is highly compartmentalized in cytoplasmic structures that resemble the ER since there is a high degree of colocalization as shown in merged image. B) Manders´ coefficient indicates that 45% of Mag-Fluo-4 signal colocalizes with the ER (n = 26 cells from 4 different experiments). C) Representative image of cells transfected with mCherry-β4-GalT1 (mCherry-Golgi-7) as indicator of the Golgi apparatus and loaded with Mag-fluo-4. Both the ER and Golgi region were stained with Mag-fluo-4. D) Manders´ coefficient indicates that 30 % of the Mag-Fluo-4 signal colocalized with Golgi (n = 33 cells from 3 independent experiments). Note that the nucleoplasm is void of fluorescence but the nuclear envelope is evident with Mag-fluo-4. Under our loading conditions, 75 % of Mag-Fluo-4 fluorescence signal reflects luminal [Ca^{2+}] in both the ER and the Golgi apparatus. Manders´ data are the mean ± SEM for the number of cells indicated. The scale bar is 5 μm.

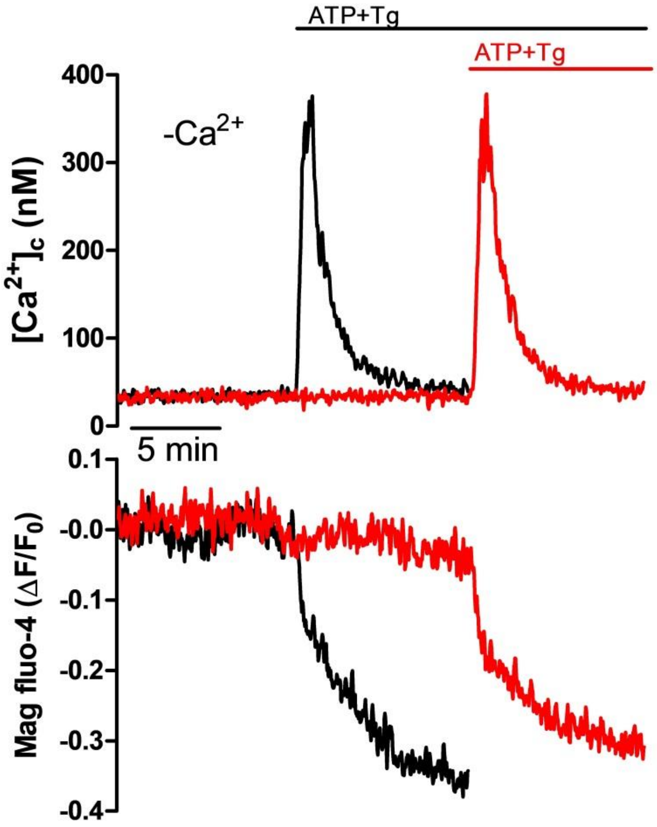

Supplementary Fig. 2 Increasing the time that cells were in the absence of external [Ca^{2+}] (0.1 mM EGTA) did not modify the [Ca^{2+}]$_c$ and [Ca^{2+}]$_L$ responses induced by ATP and Tg. HeLa cells were kept for either 10 (black trace) or 20 min (red trace) in the absence of external [Ca^{2+}] (saline solution without added Ca^{2+} and supplemented with 0.1 mM EGTA (-Ca^{2+}) and the combination of ATP and Tg was applied at the indicated times. Notice that both the peak [Ca^{2+}]$_c$ and the nadir of the [Ca^{2+}]$_L$ responses were basically the same, arguing that the extra time that cells were in the absence of external [Ca^{2+}] did not decrease the amplitude of both type of responses. These traces are representative of 3 independent experiments.

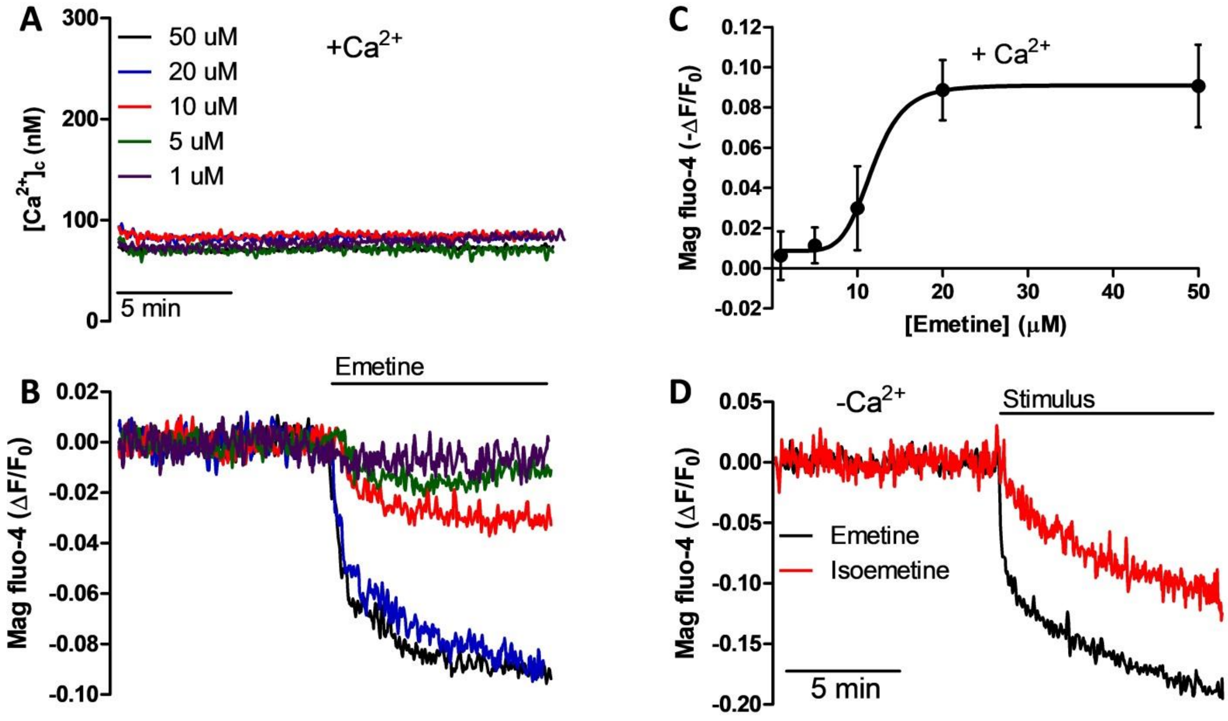

Supplementary Fig. 3 Emetine and isoemetine reduce the luminal [Ca^{2+}] in HeLa cell populations. A) Average traces of [Ca^{2+}]$_c$ in response to different concentrations of emetine. B) The same cells shown in A) except that this is the [Ca^{2+}]$_L$ response induced by emetine. These data were obtained in the presence of 1.8 mM [Ca^{2+}] (+Ca^{2+}). C) Concentration-response curve for emetine-induced reduction of the [Ca^{2+}]$_L$ using data shown in panel B). The EC_{50} was 11.4 µM with an extremely steep Hill coefficient (6.9). This high Hill coefficient suggests that the presence of external Ca^{2+} was opposing to the Ca^{2+} releasing activity of emetine. D) Mag-Fluo-4 fluorescence response to the application of either emetine (black trace) or isoemetine (red trace) in the absence of external [Ca^{2+}] (-Ca^{2+}). Data shown are the mean ± SEM of n = 5 independent experiments.

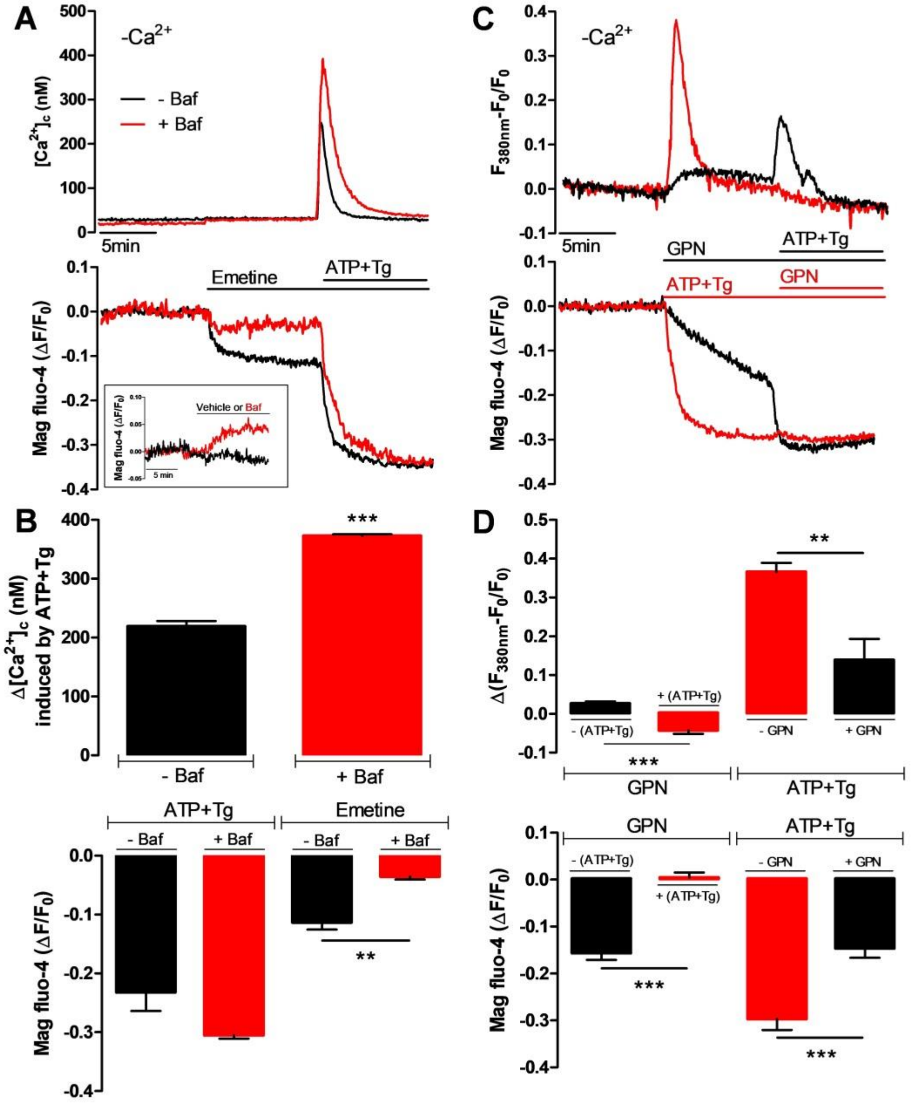

Supplementary Fig. 4 Emetine released Ca^{2+} from a bafilomycin-sensitive compartment and GPN inhibited agonist-induced Ca^{2+} release from the ER. A) Average traces of simultaneous recordings of $[Ca^{2+}]_c$ and $[Ca^{2+}]_L$ in the absence of external $[Ca^{2+}]$ and in presence of EGTA 0.1 mM ($-Ca^{2+}$) for cells that were pretreated with bafilomycin (baf) for 15 min before the application of emetine (red trace) when compared to the control response (black trace). Inset shows that baf application clearly increased the $[Ca^{2+}]_L$. B) Average peak $[Ca^{2+}]_c$ response induced by ATP and Tg was significantly increased by baf (red bar, n = 3) when compared to control response (black bar, n = 8) C) Average changes in 380 nm Fura-2 fluorescence (because 340/380 ratio was altered by GPN due to changes in the 340 nm fluorescence) that indicates modifications in the $[Ca^{2+}]_c$ (top) and $[Ca^{2+}]_L$ (bottom) in those cells that were exposed to GPN, where indicated; either before (black trace) or after the addition of ATP and Tg (red trace). D) Upper panel shows that the $[Ca^{2+}]_c$ level was increased by GPN but only when added before (left black bar) and not after the combination of ATP and Tg (left red bar). Additionally, GPN decreased the ATP and Tg-induced increase of the $[Ca^{2+}]_c$ (right black bar, n=7) when compared with control response (right red bar, n = 6). Data are presented as mean ± SEM of n number of independent experiments.

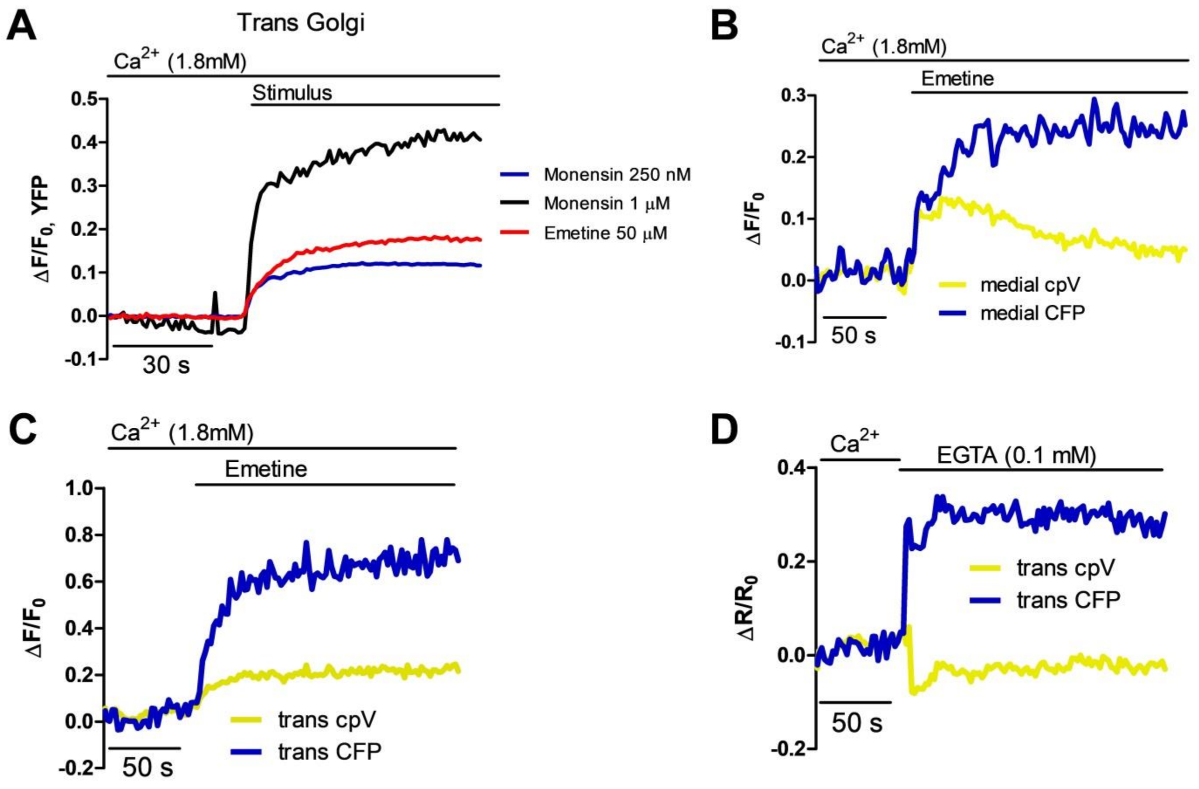

Supplementary Fig. 5 Emetine increases the luminal pH and decreases the $[Ca^{2+}]_L$ in Golgi apparatus of HeLa cells. A) HeLa cells transfected with Go-D1cpv where only Venus (YFP) was excited to observe pH effects instead. The application of either monensin (250 nM or 1 μM) or emetine (50 μM) increased fluorescence reflecting an elevation of pH in the trans-Golgi region. Note that 250 nM monensin (blue trace, n = 18 cells) produced a similar increase in fluorescence as 50 μM emetine (red trace, n = 32 cells), which was much smaller than the one observed with 1 μM monensin (black trace, n = 28 cells). HeLa cells expressing either B) medialGo-D1cpv (n = 12 cells) or C) Go-D1cpv (n = 23 cells) were excited to detect FRET but instead of showing the ratio (520/480), the individual changes in fluorescence for both Cyan (CFP, blue trace) and Venus (cpV, yellow trace) in response to emetine (50 μM) are displayed. Note that initially both 480 nm and 520 nm fluorescence signals increased suggesting that pH was increased and the $[Ca^{2+}]_L$ was reduced, later on, the main effect of emetine was reduction of the $[Ca^{2+}]_L$. D) Average recording of both FRET signals for Go-D1cpv probe in response to 0.1 mM EGTA perfusion to decrease the external $[Ca^{2+}]$. This is an example of pure reduction of the trans-Golgi $[Ca^{2+}]_L$ as fluorescence signals moved in opposite directions (n = 19 cells).
